# Fruquintinib-induced renal-limited thrombotic microangiopathy: a case report

**DOI:** 10.1186/s12882-024-03598-8

**Published:** 2024-05-18

**Authors:** Ruiping Zhao, Ruichen Fan, Yan Pan, Yuze Han, Ying Wang, Weidong Chen

**Affiliations:** 1 Department of Nephrology, the First Affiliated Hospital of Bengbu Medical University, Bengbu, China; 2 Department of Pharmacy, the First Affiliated Hospital of Bengbu Medical University, Bengbu, China

**Keywords:** Fuquinitinib, Thrombotic microangiopathy, Renal restriction

## Abstract

**Background:**

Fruquintinib is a highly selective inhibitor of vascular endothelial growth factor receptor (VEGFR). Currently, there are no reported cases of fruquintinib causing kidney-restrictive thrombotic microangiopathy (TMA) in the available Chinese and foreign literature.

**Case presentation:**

In this case report, we presented a 73-year-old patient receiving fruquintinib for metastatic colon cancer, manifesting abundant proteinuria, in which kidney-restrictive TMA was also diagnosed through renal biopsy. As far as we were concerned, this was the frst reported in terms of fruquintinib-induced kidney-restrictive TMA confrmed by renal biopsy.

**Conclusion:**

This case indicates that fruquintinib may result in kidney-restrictive TMA, which is a rare but life-threatening complication of cancer treatment drug. Therefore, regular monitoring of proteinuria and blood pressure is imperative for all patients undergoing anti-VEGF drug therapy. And renal biopsy should be promptly conducted to facilitate early detection of thrombotic microangiopathy.

## Background

Vascular endothelial growth factor (VEGF) is crucial for the growth and proliferation of tumor cells, thus blocking the pathway of vascular endothelial cell growth factor has anti-tumor effects. Fruquintinib is a highly selective inhibitor of vascular endothelial growth factor receptor (VEGFR) independently developed by Hutchison China MediTech Limited (Chi-Med), which inhibits the activity of VEGFR kinase, suppresses the phosphorylation of VEGFR2/3, leads to the proliferation of vascular endothelial cells and luminal formation, thereby inhibiting tumor angiogenesis and achieving anti-tumor proliferation effects.

Oral administration of fruquintinib can lead to adverse effects such as hypertension, hand-foot skin reactions, proteinuria, difficulty in speaking, diarrhea, rash, and other reactions. Currently, there are no reported cases of fruquintinib causing kidney-restrictive thrombotic microangiopathy (TMA) in the available Chinese and foreign literature. In order to address this research gap, this article presents a case of a patient with metastatic rectal cancer who developed nephrotic syndrome due to TMA after receiving oral fruquintinib, thereby offering valuable insights for the future administration of this drug.

## Case presentation

A 73-year-old male patient received a diagnosis of rectal adenocarcinoma accompanied by multiple liver metastases two years ago. Genetic testing revealed a KRAS mutation rate of 65.8%. Following the exclusion of contraindications, he initiated a targeted combination chemotherapy comprising Bevacizumab and XELOX (oxaliplatin, capecitabine) for six cycles. Subsequently, the regimen was modified to Bevacizumab combined with capecitabine (D1-14) for a total of three cycles upon stabilization of the disease.

After one year, the patient experienced a reduction in tumor lesions and subsequently underwent laparoscopic radical resection of rectal cancer (Dixon procedure) in the Department of Gastrointestinal Surgery. Following the surgery, the patient received four cycles of chemotherapy comprising bevacizumab in combination with capecitabine and oxaliplatin. However, assessment revealed progression of the primary disease, prompting a modification in the treatment regimen to include bevacizumab, capecitabine, and fruquintinib. Prior to initiating fruquintinib, the patient's serum albumin level was 41.3 g/L, with negative results for urine protein and red blood cells, normal kidney function, and no hypertension. Subsequently, with the introduction of fruquintinib, the patient experienced adverse effects such as hand and foot skin peeling, elevated blood pressure, increased urine foam, and bilateral lower limb edema. A month later, the patient sought consultation at our hospital. Laboratory findings revealed significant changes, including 3 + urine protein, a decrease in blood albumin to 24.4 g/L, and an increase in creatinine levels to 113 µmol/L. Despite a three-month reduction in fruquintinib dosage, the patient continued to exhibit persistent proteinuria, clinically indicative of nephrotic syndrome with mild elevation of blood creatinine. Following consultation with nephrology specialists, the patient was transferred to the nephrology department for further management.

**Fig. 1:**
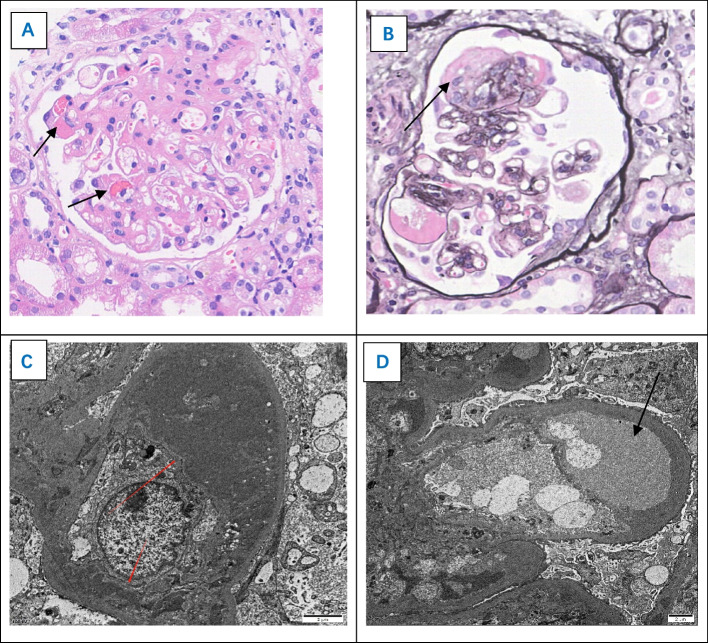
Pathological biopsy of glomerulus showing thrombotic microangiopathy. **A** Hematoxylin–eosin staining × 400. **B** Periodic acid silver methenamine stain × 400. **C**, **D** Electron microscope. Electron microscopy **C** revealed mesangial insertion beneath the endothelium of the capillary, resembling membranoproliferative glomerulonephritis; Electron microscopy **D** revealed a loose subendothelial space with regional widening of the glomerular basement membrane

The patient underwent comprehensive laboratory examinations upon admission. The results revealed a blood albumin level of 17.1 g/L, creatinine level of 113umol/L, negative anti-phospholipase A2 receptor (PLA2R) antibodies, negative Coombs test, absence of fragmented red blood cells on blood smear, normal serum bilirubin, and elevated lactate dehydrogenase. Additionally, the urine analysis showed proteinuria of 3 + with 95 red blood cells/µl, and a 24-h urine protein quantification of 9.41 g. Secondary factors such as autoimmune diseases, bacterial, and viral infections were ruled out. Subsequently, a renal biopsy was performed, and the postoperative pathology (see Fig. 1) revealed the following findings: Immunofluorescence showed negativity for IgG, IgA, IgM, C3, C1q, Kappa, lambda, PLA2R, and THSD7A. Electron microscopy displayed a loose subendothelial space with regional widening of the glomerular basement membrane, approximately 60% foot process fusion, and electron-dense deposits are observed beneath the endothelium within the glomerular mesangial area and the basal membrane of segmental capillary loops. Light microscopy exhibited thrombus formation within the glomerular capillary loops, widening of the mesangial area with several areas of mesangial dissolution, and segmental changes resembling membranoproliferative glomerulonephritis (MPGN). The combined results of immunofluorescence, electron microscopy, and light microscopy are consistent with anti-VEGF drug-related kidney injury—thrombotic microangiopathy.

**Fig. 2:**
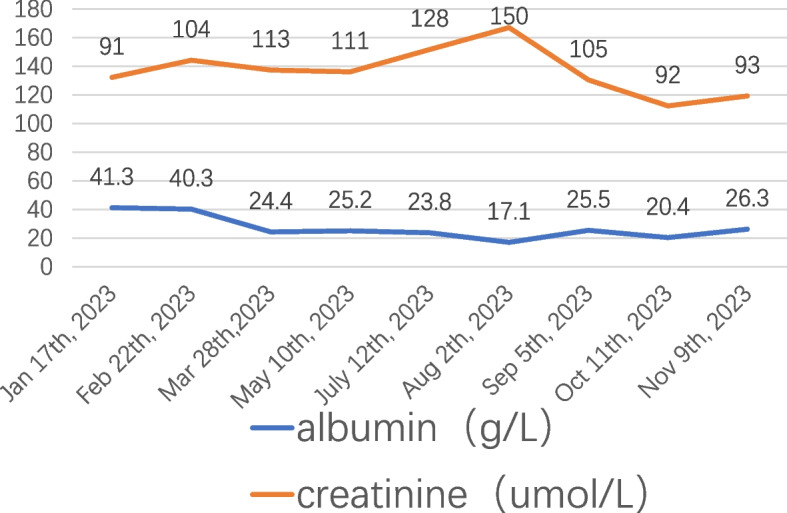
The laboratory indicators of the patient

The physician requested a multidisciplinary consultation for the patient, involving the departments of oncology, pharmacy, and nephrology. Following the consultation, it was recommended to discontinue fruquintinib and initiate Sacubitril/Valsartan at a dosage of 200 mg/day. Additionally, the chemotherapy regimen was modified to include Tiragolumab alongside Capecitabine. The patient has been off Fruquintinib for 5 months, experiencing reduced edema, stabilized blood pressure, with serum albumin levels at 26.3 g, creatinine levels at 93 micromoles/liter, and urine protein at 2 + . A summary of the patient's laboratory indicators is presented in Fig. 2.

## Discussion and conclusion

The patient had previously undergone treatment with VEGFR inhibitors, including bevacizumab. However, during follow-up, there were no indications of proteinuria or hematuria, and renal function remained within normal limits. Due to disease progression, furqinitinib was introduced to the treatment protocol. Approximately two weeks after initiating furqinitinib therapy, the patient developed hand-foot skin reactions, hypertension, proteinuria, and other adverse effects, clinically manifesting as nephrotic syndrome. There were no systemic manifestations of TMA, and both medical history and laboratory analyses did not support diagnoses of thrombotic thrombocytopenic purpura (TTP) or hemolytic uremic syndrome (HUS). Renal pathology suggested thrombotic microangiopathy induced by anti-VEGF medication. Given the temporal relationship and direct association with furqinitinib use, the patient's renal impairment was attributed to furqinitinib-induced renal-limited thrombotic microangiopathy.

Thrombotic microangiopathy presents as a pathological syndrome featuring microangiopathic hemolytic anemia, thrombocytopenia, organ involvement, and dysfunction. Its etiology encompasses thrombotic thrombocytopenic purpura and hemolytic uremic syndrome [1]. Additionally, secondary thrombotic microangiopathy can arise from various factors, including infection, autoimmune diseases, solid organ transplantation, radiation, disseminated malignancies, and anti-tumor drugs.

TMA induced by anti-tumor drugs can be categorized into two types: Type I TMA, induced by anticancer drugs, encompasses all chemotherapy regimens (such as Streptomyces C, Gefitinib, etc.), which potentially contribute to long-term kidney damage, increased incidence, and mortality rates. Type II TMA, induced by anti-tumor drugs, includes anti-VEGF drugs. VEGF plays a crucial role in maintaining the physiological function of glomerular endothelial cells, podocytes, and renal tubular epithelial cells. Excessive or deficient VEGF signaling has been shown to negatively impact both the structure and function of podocytes [2]. Within podocytes, VEGF signaling plays a crucial role in organizing the actin cytoskeleton. Proper signaling also provides a trophic survival signal via Akt (protein kinase B) and regulates cell cycle function through Ras/Raf interactions. Additionally, appropriate VEGF stimulation suppresses Nuclear Factor Kappa-light-chain-enhancer of activated B cells-mediated targets of inflammation and the activation of the renin–angiotensin–aldosterone system. In endothelial cells, VEGF signaling contributes to nitric oxide production and vasodilation, offering a trophic signal for endothelial survival and function. Furthermore, VEGF signaling regulates Di-Acyl Glycerol-Kinase-Epsilon, and its disruption can lead to thrombotic microangiopathy. Consequently, significant inhibition of this pathway can result in podocyte effacement, inflammation, and nephrotic syndrome by disrupting the podocyte cytoskeleton. Moreover, there is a clear association with thrombotic disorders and hypertension, stemming from endothelial cell dysfunction, dysregulation of clotting, and disruption of nitric oxide synthesis [3].

The kidney diseases associated with VEGF pathway inhibitors commonly manifest in two pathological categories: thrombotic microangiopathy, confined to renal tissues and devoid of abnormal ADMATS13 activity or complement gene mutations [4], and a subset of patients experience amelioration upon drug cessation. Angiotensin-converting enzyme inhibitors serve as the primary therapy for kidney-restricted TMA; however, efficacy is limited in cases with substantial proteinuria within the nephrotic syndrome spectrum. Marco Stortz [5] et al. documented a case of kidney-restricted TMA induced by bevacizumab monoclonal antibody, resulting in systemic TMA subsequently alleviated by steroid pulse therapy and plasma exchange. Rituximab administration has shown promise in relieving proteinuria in non-responsive patients to drug discontinuation [6]. The patient presented with hypertension preceding proteinuria, and the blood pressure once reached as high as 200/120 mmHg, with negative results for renal pathological immunofluorescence, multiple serum autoantibodies, and complement levels, ruling out immunologically mediated TMA at present.In light of hepatoprotection, glucocorticoids and biologics were not pursued. Following fruquintinib cessation and sacubitril/valsartan addition, serum albumin increment, urine protein reduction, serum creatinine normalization, edema alleviation, and renal function improvement were observed at the 5-month follow-up. It is hypothesized that furquidone-induced malignant hypertension may result in elevated mechanical shear forces, resulting in endothelial damage and rupture of the glomerular capillaries in the kidneys. This pathological process presents as capillary endothelium loosening, substantial widening of the affected area, and segmental changes resembling MPGN. Concurrently, activation of the renin-angiotensin system contributes to the development of TMA.Necessitating further investigation into complement pathway activation, cell-mediated immunity, and other mechanisms. Additionally, podocyte diseases, encompassing Minimal change disease (MCD) and focal segmental glomerulosclerosis (FSGS), represent another TMA variant induced by antitumor drugs. Receptor tyrosine kinase inhibitors (RTKIs) inhibit RelA activity, potentially leading to increased c-mip expression in podocytes, which disrupts the podocyte cytoskeleton and foot process fusion [7]. Hence, RTKIs predominantly instigate MCD or FSGS in certain patients. Clinical presentations encompass substantial proteinuria, potentially progressing to nephrotic syndrome, with a majority of patients typically experiencing amelioration post cessation of the medication. The renal pathology of this patient revealed podocyte fusion of nearly 60%, which was related to the use of anti-VEGF drugs. Despite not meeting the diagnostic criteria for podocytopathy, the impairment of the glomerular filtration barrier can precipitate substantial proteinuria. Moreover, there have been documented instances of VEGF pathway inhibitors provoking acute interstitial nephritis, acute tubular necrosis, crescentic glomerulonephritis, and proliferative immune complex glomerulonephritis [8].

Fruquintinib, a VEGFR inhibitor independently developed in China and approved by the CFDA, exhibits potent and highly selective inhibitory activity against all three isoforms of VEGFR (VEGFR-1, 2, 3), thus concomitantly suppressing tumor angiogenesis and lymphangiogenesis. As a third-line therapy for metastatic colorectal cancer, it significantly extends patients' survival duration [9]. Notably, fruquintinib demonstrates pharmacological efficacy at lower blood concentrations compared to other VEGFR inhibitors, thereby enhancing safety profiles. However, akin to other VEGFR inhibitors, hypertension and proteinuria represent common adverse reactions to fruquintinib. Eremina et al. [10] documented six cases of renal-limited thrombotic microangiopathy induced by bevacizumab usage. Upon cessation of the medication, improvements were observed in blood pressure, proteinuria, and renal function, suggesting the reversibility of these bevacizumab-induced side effects. Nonetheless, not all patients experience such favorable outcomes. Prolonged and substantial proteinuria may lead to irreversible renal damage, making medication discontinuation sometimes the most prudent course of action. For individuals who fail to respond to discontinuation, treatment modalities such as glucocorticoids, rituximab, and plasma exchange may be considered; however, the efficacy of these interventions remains uncertain [11].

Therefore, when employing anti-VEGF medications in clinical practice, it is crucial to acknowledge the role of VEGF as a pivotal signaling molecule synthesized by podocytes. These medications commonly induce proteinuria and hypertension as adverse effects. Therefore, regular monitoring of proteinuria and blood pressure is imperative for all patients undergoing anti-VEGF drug therapy. Prompt cessation of the drug is warranted if there is onset or progressive aggravation of hypertension, hematuria, proteinuria, etc. Additionally, if circumstances permit, renal biopsy should be promptly conducted to facilitate early detection of thrombotic microangiopathy. Furthermore, enhancing communication and collaboration among multidisciplinary teams including oncology, nephrology, and pharmacy is essential to actively advance the interdisciplinary realm of tumor nephropathy. This approach aims to foster early detection and timely management of nephrotoxicity induced by antitumor medications, thereby ameliorating the prognosis of malignant tumor patients and augmenting their quality of life.

## Data Availability

The datasets used in this study are available from the corresponding author on reasonable request.

## References

[1] Mazzierli T, Allegretta F, Maffini E (2023). Drug-induced thrombotic microangiopathy: an updated review of causative drugs, pathophysiology, and management. Front Pharmacol.

[2] Hanna RM, Barsoum M, Arman F (2019). Nephrotoxicity induced by intravitreal vascular endothelial growth factor inhibitors: emerging evidence. Kidney Int.

[3] Shye M, Hanna RM, Patel SS (2020). Worsening proteinuria and renal function after intravitreal vascular endothelial growth factor blockade for diabetic proliferative retinopathy. Clin Kidney J.

[4] Yin Q, Guo N, Zhou X (2022). Regorafenib-induced renal-limited thrombotic microangiopathy: a case report and review of literatures. BMC Nephrol.

[5] Stortz M, Shmanko K, Kraus D (2023). Plasma exchange for treatment of a therapy-related thrombotic microangiopathy in a patient with advanced hepatocellular carcinoma—A case report. Clin Case Rep.

[6] Gourley BL, Mesa H, Gupta P (2010). Rapid and complete resolution of chemotherapy-induced thrombotic thrombocytopenic purpura/hemolytic uremic syndrome (TTP/HUS) with rituximab. Cancer Chemother Pharmacol.

[7] Zhang SY, Fan Q, Moktefi A (2021). CMIP interacts with WT1 and targets it on the proteasome degradation pathway. Clin Transl Med.

[8] Hanna RM, Tran NT, Patel SS (2020). Thrombotic microangiopathy and acute kidney injury induced after intravitreal injection of vascular endothelial growth factor inhibitors VEGF blockade-related TMA after intravitreal use. Front Med.

[9] Li J, Qin S, Xu RH (2018). Effect of fruquintinib vs placebo on overall s urvival in patients with previously treated metastatic colorectal cancer: the FRE SCO randomized clinical trial. JAMA.

[10] Eremina V, Jefferson JA, Kowalewska J (2008). VEGF inhibition and renal thrombotic microangiopathy. N Engl J Med.

[11] Izzedine H, Perazella MA (2015). Thrombotic microangiopathy, cancer, and cancer drugs. Am J Kidney Dis.

